# LncRNA HAND2‐AS1 represses cervical cancer progression by interaction with transcription factor E2F4 at the promoter of C16orf74

**DOI:** 10.1111/jcmm.15117

**Published:** 2020-04-21

**Authors:** Junling Gong, Haiying Fan, Jing Deng, Qiumei Zhang

**Affiliations:** ^1^ Department of Obstetrics and Gynecology Linyi People's Hospital Linyi China; ^2^ Hemodialysis Room Linyi People's Hospital Linyi China; ^3^ Department of Internal Medicine Miaoshan Health Center Linyi China

**Keywords:** C16orf74, cervical cancer, E2F4, HAND2‐AS1, invasion, migration, proliferation

## Abstract

Cervical cancer is one of the major malignancies, the pathophysiology and progression of which remain to be scarcely understood. Long non‐coding RNAs (lncRNAs) have been previously implicated in the progression of cervical cancer. Here, the purpose of this study was to identify whether lncRNA heart‐ and neural crest derivative‐expressed 2‐antisense RNA 1 (HAND2‐AS1) affect the development of cervical cancer through regulation of chromosome 16 open reading frame 74 (C16orf74) by mediating a transcription factor E2F4. RT‐qPCR was performed to determine the expression of HAND2‐AS1 in cervical cancer cells. Then, cervical cancer cells were treated with HAND2‐AS1 or si‐E2F4 RNA, or C16orf74, after which the proliferation, colony formation, migration and invasion were detected. Moreover, the binding between HAND2‐AS1 and E2F4 or between E2F4 and C16orf74 was explored. Finally, the tumorigenesis of cervical cancer cells was measured in nude mice with altered HAND2‐AS1/E2F4/C16orf74 expression. HAND2‐AS1 exhibited poor expression in cervical cancer, and HAND2‐AS1 overexpression suppressed the proliferation, colony formation, migration and invasion of cervical cancer cells. In addition, HAND2‐AS1 was found to recruit transcription factor E2F4 to C16orf74 promoter region and down‐regulate C16orf74 expression. Lastly, HAND2‐AS1/E2F4/C16orf74 modulated the tumorigenesis of cervical cancer in nude mice. In conclusion, this study provided evidence on the inhibitory effect of HAND2‐AS1 on the development of cervical cancer through the suppression of C16orf74 expression by recruiting transcription factor E2F4. This study highlights the potential of lncRNA HAND2‐AS1 as a target in the treatment of cervical cancer.

## 1 INTRODUCTION

Cervical cancer is the fourth‐most prevalent cancer in women, characterized by high morbidity and mortality.^1^ It is considered to be induced by high‐risk human papillomavirus (HPV) persistence in condition of immunosuppressive tumour microenvironment.^2^ Previous studies have reported that HPV vaccines and early diagnosis through regular cervical screening could be beneficial in the prevention of cervical cancer.^3^, ^4^ Tumour necrosis factor‐α immunotherapy has also been found to lessen the risk of cervical cancer development.^5^ Despite the current breakthroughs, the survival rate of cervical cancer remains low in cases of recurrent metastatic cervical cancer due to the limited treatment options,^6^, ^7^ making the finding of more effective therapies for cervical cancer an urgent demand.

Long non‐coding RNAs (lncRNAs) have been regarded as crucial factors in tumorigenesis of different several kinds of cancer, including cervical cancer.^8^, ^9^ LncRNAs, which range from 200 nt to 100 kb in length, could function as either oncogenic or tumour‐suppressive factors due to their diverse regulatory mechanisms.^10^ LncRNA heart‐ and neural crest derivative‐expressed protein 2 (HAND2)‐AS1 has been proven to inhibit tumours in various malignant tumours.^11^ In addition, there is an aberrant expression of HAND2‐AS1 in HPV‐negative head and neck squamous cell carcinoma.^12^ More recently, the down‐regulation of HAND2‐AS1 has been reported to be capable of the distinguishing cervical squamous cell carcinoma patients from healthy controls.^13^ Nevertheless, the underlying regulatory mechanism of HAND2‐AS1 has not been thoroughly studied in cervical cancer.

E2F transcription factor family members play crucial roles in the regulation of cell cycle, cell differentiation, DNA repair and cell death.^14^ According to a previous study, E2F4 participates in the regulation of transcription of multiple core genes in the tumorigenesis of Burkitt lymphoma^15^ and cervical cancer.^16^ Chromosome 16 open reading frame 74 (C16orf74), also known as MGC17624, is a gene locus on chromosome 16q24.1, whose function requires further elucidation.^17^ C16orf74 is involved in the mediation of the proliferation and invasion of pancreatic ductal adenocarcinoma (PDAC), making it a promising target for PDAC treatment.^18^ Most importantly, lncRNA GAS5 regulates the transcription of EZH2 by recruiting E2F4 to EZH2 promoter, as demonstrated in another study.^19^ The aforementioned evidence led to the hypothesis that lncRNA HAND2‐AS1 might affect the progression of cervical cancer through the regulation of C16orf74 expression by recruiting the transcription factor E2F4.

## 2 METHODS AND MATERIALS

### 2.1 Ethics statement

The study was conducted with the approval of the Ethics Committee of Linyi People's Hospital. Written informed consents were obtained from all patients and their guardians prior to the study. All animal experiments were carried out in accordance with the principles and procedures of Guide for the Care and Use of Laboratory Animal by the National Institutes of Health.

### 2.2 Microarray analysis

The cervical cancer‐related gene expression data set (GSE63678) as well as the annotation probe file was detected using the [HG‐U133A_2] Affymetrix Human Genome U133A 2.0 Array and downloaded from Gene Expression Omnibus database (https://www.ncbi.nlm.nih.gov/geo/). The Affy package of R language software was used for data background correction and normalization processing.^20^ Next, the non‐specific filtration for gene expression data set was performed using the linear models and empirical Bayes methods in Limma package combined with the traditional *t* test. Finally, the Benjamini‐Hochberg false discovery rate (FDR) was employed for multiple‐testing correction, and the differentially expressed lncRNAs and mRNAs were screened out with the threshold set at FDR < 0.05 and absolute fold change ≥1.5.^21^ Lastly, the co‐expression relationship between HAND2‐AS1 and C16orf74 in The Cancer Genome Atlas‐Cervical Cancer (TCGA‐CESC) data set was obtained by the StarBase database (http://starbase.sysu.edu.cn/index.php).

### 2.3 Study subjects

The cervical cancer tissues were obtained from 57 patients (aged from 39 to 71 years, with the average age of 53.98 ± 8.45 years) diagnosed with cervical cancer in Linyi People's Hospital from December 2015 to December 2016. Patients received no radiotherapy or chemotherapy prior to surgical resection. Patients who had other systemic diseases, in pregnancy or other cancer‐related diseases were excluded. Meanwhile, normal tissues were obtained from 20 patients (aged from 45 to 70 years, with the average age of 58.30 ± 7.89 years) who had undergone total hysterectomy or had no malignant lesions or precancerous lesion.

### 2.4 Follow‐up

Follow‐up was performed for 57 patients with cervical cancer to observe their overall survival (OS), which was defined as the interval between resection and death or the last follow‐up examination. The follow‐up lasted until December 2018 and was conducted by returning visit or telephone calls. Within the 3 years, a total of 6 patients out of the 57 patients were lost in the follow‐up (follow‐up rate of 89.47%). The follow‐up period ranged between 5 and 36 months.

### 2.5 Cell treatment

The human cervical epithelial immortalized cell H8 and 4 cervical cancer cells (Caski, HeLa, Siha and HCE1) were obtained from American Type Culture Collection (Manassas, VA, USA), followed by culturing in Royal Park Memorial Institute 1640 medium containing 10% foetal bovine serum (FBS), 100 U/mL penicillin and 100 μg/mL streptomycin. The sequence of HAND2‐AS1 or C16orf74 cDNA as well as the control sequence was ligated to the PLV‐Neo vector (Inovogen Tech. Co.), and E2F4 shRNA sequence and the control sequence were ligated to the PLKO‐Puro vector (Sigma‐Aldrich, SF), respectively. The plasmids were cotransfected with psPAX2 and pMD2.G (Addgene) into HEK293T cells.

### 2.6 RNA isolation and quantitation

By using TRIzol (15596026, Invitrogen), total RNA was extracted and then reversely transcribed into complementary DNA (cDNA) by a reverse transcription kit (RR047A, Takara). Next, RT‐qPCR was carried out using the SYBR Premix EX Taq kit (RR420A, Takara) on the ABI 7500 PCR instrument (Applied Biosystems). Shanghai Sangon Biotechnology Co., Ltd. was commissioned to synthesize the primers (Table 1). The Ct value of each well was recorded. β‐Actin was used as the internal reference, and the relative expression of target genes between the experiment group and the control group was detected using the 2−ΔΔCt method.^22^ The experiments were performed in triplicate.

**Table 1:** Primer sequences for RT‐qPCR

Genes	Primer sequence (5′‐3′)
HAND2‐AS1	F: GGAGTCACAGGCAGTCGTAGA
	R: GAAGGCACAGATCATTCATGG
β‐Actin	F: GGCGACGAGGCCCAGA
	R: CGATTTCCCGCTCGGC
C16orf74	F: CCGGAATTCGACATGGGGCTTAAGATGTCC
	R: CCGCTCGAGGGCTTCTGGGTCGATTTCTCC

Abbreviations: C16orf74, chromosome 16 open reading frame 74; F, forward; HAND2‐AS1, heart and neural crest derivative‐expressed 2‐antisense RNA 1; R, reverse; RT‐qPCR, reverse transcription quantitative‐polymerase chain reaction.

### 2.7 Fluorescence in situ hybridization (FISH) assay

Fluorescence in situ hybridization assay was used to determine the subcellular localization of HAND2‐AS1 in cervical cancer cells. In accordance with the introductions of RiboTM lncRNA FISH probe Mix (Red) (Guangzhou RiboBio Co., Ltd.), the cervical cancer cells were seeded on the cover slips in the 6‐well plate. After 1 day, upon reaching 80% confluence, the cells were fixed by 1 mL 4% paraformaldehyde, treated with 2 μg/mL protease K, glycine and ethyl phthalate reagent, followed by an incubation with 250 μL pre‐hybridization solution at 42°C for 1 hour. Next, the cells underwent incubation overnight at 42°C with 250 μL hybridization solution containing probe (300 ng/mL). Subsequently, cells were further incubated with 4′6‐diamidino‐2‐phenylindole diluted with phosphate buffer saline‐Tween 20 at a ratio of 1:800 for 5 minutes in a 24‐well plate. Finally, the cells were mounted by anti‐fluorescence quencher, observed and photographed under a fluorescence microscope (Olympus Optical Co., Ltd) with 5 different visual fields selected.

### 2.8 RNA immunoprecipitation (RIP)

The relationship between HAND2‐AS1 and E2F4 protein was examined with the use of the RIP kit (Millipore Co., Ltd). The cervical cancer cells were lysed in RIP assay buffer (P0013B, Beyotime Biotechnology Co.) for 5 minutes, followed by a centrifugation at 12 000 *g* for 10 minutes at 4°C, with the supernatant collected. Next, RIP buffer containing magnetic beads coated with E2F4 antibody (sc‐6851, 2 µg per 1 mL of cell lysate, Santa Cruz Biotechnology, Inc) or negative control (NC) immunoglobulin G (IgG) antibody (ab172730, 1:100, Abcam) was added to the extracts and incubation was carried out at 4°C overnight. Subsequently, the magnetic bead‐immunoprecipitated complex was washed with 900 μL RIP Wash Buffer. Finally, the input and immunoprecipitated complex were treated by protease K and RNA was extracted for subsequent PCR detection.

### 2.9 Chromatin immunoprecipitation (ChIP) assay

The cervical cancer cells were fixed with formaldehyde for 10 minutes. The ultrasonic breaker was set to 10 seconds per ultrasonic cycle with 10‐second intervals with 15 cycles to break the chromatin. Subsequently, the products were centrifuged at 12 000 *g* for 10 minutes at 4°C to collect supernatant, which was divided into two tubes and incubated with NC mouse antibody to IgG (ab172730, 1:100, Abcam) or antibody to E2F4 (sc‐6851, 2 µg per 1 mL of cell lysate, Santa Cruz Biotechnology, Inc) overnight at 4°C. Subsequently, DNA‐protein complex was precipitated using Protein Agarose/Sepharose, and centrifugation was carried out at 12 000 *g* for 5 minutes at 4°C. Afterwards, the cross‐linking was reversed overnight at 65°C, and DNA fragments were recovered following extraction and purification by phenol/chloroform. Lastly, reverse transcription quantitative‐polymerase chain reaction (RT‐qPCR) was conducted to detect the binding of E2F4 with C16orf74 promoter region with the C16orf74‐specific primer.

### 2.10 Dual‐luciferase reporter gene assay

The binding sites between C16orf74 promoter region and E2F4 were analysed using online predication. The recombinant vectors C16orf74‐wild‐type (WT) or C16orf74‐mutant type (MUT) were transfected into HEK293T cells (Shanghai Beinuo Biotechnology Co., Ltd.) with si‐NC and si‐E2F4, respectively. Following a 48‐hour transfection, the cell lysates were collected and the Firefly Luciferase Reporter Gene Assay Kit (RG005, Beyotime Biotechnology Co.) was used to detect the firefly and Renilla luciferase activities on a microplate reader (MK3, Thermo Fisher Scientific) at 560 nm.

### 2.11 Western blot analysis

The tissue or cells were lysed by being placed on ice for 10 minutes, followed by the protein quantification using a bicinchoninic acid assay protein quantitative kit (MultiScience (LIANKE) Biotech, Co., Ltd.). Next, the proteins were loaded and separated on sodium dodecyl sulphate‐polyacrylamide gels and transferred onto a nitrocellulose membrane. Subsequently, the membrane was blocked with 5% bovine serum albumin/Tris‐buffered saline Tween (TBST) for 60 minutes and incubated at 4°C overnight with primary antibodies to N‐cadherin (ab76057, 1:1000), vimentin (ab137321, 1:2000), matrix metallopeptidase (MMP)‐9 (ab38898, 1:1000), β‐actin (ab8227, 1:2000) and MMP‐2 (ab92536, 1:2000). Afterwards, the membranes were further incubated with secondary antibody, horseradish peroxidase‐labelled goat anti‐rabbit antibody to IgG (ab6721, 1:2000) for 120 minutes at room temperature and visualized using an electrochemiluminescence kit. All antibodies were purchased from Abcam. The grey value of the corresponding protein bands was analysed using the Quantity One software, and the relative expression of protein was regarded as the ratio of grey value of target bands to that of internal reference band.

### 2.12 Cell counting kit‐8 (CCK‐8) assay

After transfection, the cells after corresponding treatment in the study were harvested and seeded into 96‐well plates (1 × 10^5^ cells/mL; 100 μL), followed by culturing overnight. Then, cell viability at 24 hours, 48 hours, 72 hours and 96 hours was determined by a CCK‐8 kit (Beyotime Biotechnology Co.). Briefly, cells in each well were added with 10 μL CCK‐8 solution and incubation carried out for 4 hours, followed by the measurement of the optical density (OD) value at 450 nm using a microplate reader.

### 2.13 Colony formation assay

The cells grown at logarithmic phase were prepared into single‐cell suspension, counted, seeded into 60‐mm culture dishes (1000 cells each dish) and incubated with CO_2_ for 14 days, with the medium renewed every 3 days. Colonies were then fixed with methanol for 15 minutes and stained for 15 minutes with crystal violet. The number of visible colonies (more than 50 cells) was counted under a microscope.

### 2.14 Transwell assay

Cells at logarithmic growth phase were starved for 24 hours, collected and suspended at the density of 2 × 10^5^ cells/mL and seeded onto the apical chamber of Transwell chamber (0.2 mL/well), and 700 μL pre‐cooled Dulbecco's modified Eagle's medium (DMEM) containing 10% FBS was added into basolateral chamber. The cells were cultured with 5% CO_2_ at 37°C for 24 hours, fixed for 30 minutes by methanol, stained for 20 minutes by 0.1% crystal violet, rinsed and air‐dried. Cells from five randomly selected fields were counted at ×100 original magnification.

Extracellular matrix (ECM) gel was diluted by serum‐free medium at a ratio of 1:8 to a final concentration of 1 mg/mL. A total of 40 μL ECM gel in the polycarbonate membrane of apical chamber underwent incubation with 5% CO_2_ at 37°C for 5 hours for polymerization. Next, the ECM gel was rehydrated by a 0.5‐hour incubation at 37°C with the pure DMEM (70 μL per chamber). The cells were starved for 24 hours, detached, centrifuged and resuspended using FBS‐free DMEM until the final concentration of cells reached 2.5 × 10^5^ cells/mL. Next, the apical chamber coated with ECM gel was added with 0.2 mL cell suspension, and the basolateral chamber was added with 700 μL pre‐cooled DMEM containing 10% FBS. Then, the following procedures were as same as the detection for cell migration above.

### 2.15 Tumour xenograft in nude mice

A total of 48 male nude mice (BALB/c, aged 4‐6 weeks, weighting 18‐22 g) purchased from Beijing HuiAo Biotechnology Co., Ltd. were maintained at 25‐27°C with 45%‐50% constant humidity. Then, in order to detect the tumorigenesis, the nude mice were injected with the cells transfected with HAND2‐AS1 alone or in the presence of sh‐NC, sh‐E2F4, oe‐NC or oe‐C16orf74. With cell concentration adjusted to 1 × 10^7^ cells/mL, 20 μL cell suspension was subcutaneously injected into the abdomen of nude mice. The tumour growth was recorded every 6 days, with tumour volume measured using the equation: *V* = *a* * *b* ^2^/2 (mm^3^), where *a* is the largest diameter, and *b* is the perpendicular diameter.^23^ After 30 days, the nude mice were killed, and tumours were collected and weighed. The tumour tissues were collected for RT‐qPCR and Western blot analysis.

### 2.16 Statistical analysis

Data analysis was performed by SPSS 22.0 statistical software (IBM Corp. Armonk, NY, USA). Measurement data were presented as mean ± standard deviation (SD). The comparison of paired data that conformed to normal distribution and homogeneity of variance was analysed using paired *t* test between two groups, and the comparison of unpaired data that conformed to normal distribution and homogeneity of variance was analysed by unpaired *t* test between two groups. The one‐way analysis of variance (ANOVA) was used to compare data among multiple groups, with Tukey's post hoc test used. The repeated‐measures ANOVA was used to compare data among multiple groups at different time‐points, and Bonferroni's post hoc test was performed. Kaplan‐Meier method was applied for calculating the OS of patients, and univariate analysis was conducted using log‐rank test. *P* < .05 was considered statistical significance.

## 3 RESULTS

### 3.1 HAND2‐AS1 is down‐regulated in the context of cervical cancer

**Figure 1:**
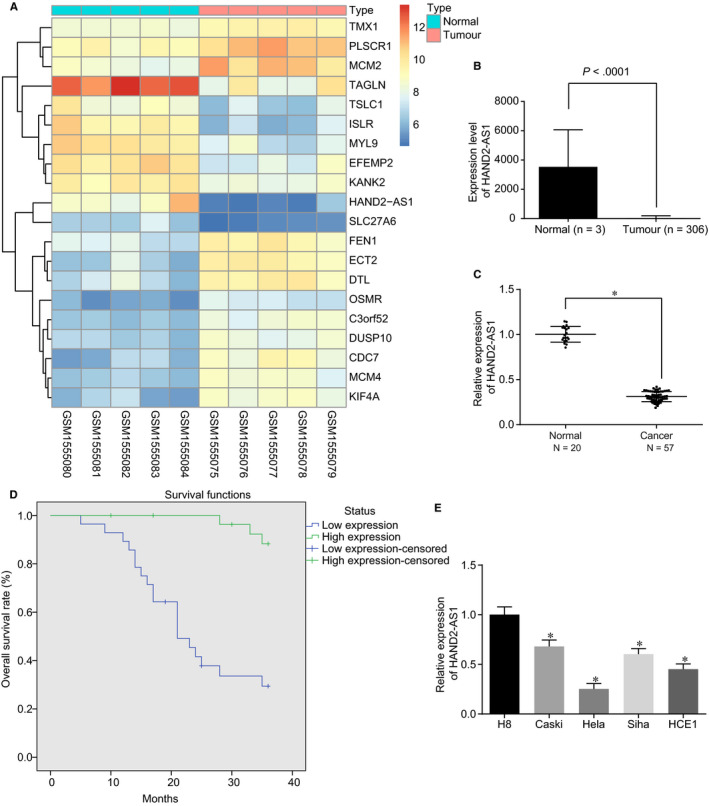
HAND2‐AS1 is down‐regulated in cervical cancer tissues and cells. A, The heatmap reveals the cervical cancer‐associated DEGs in data set GSE63678. B, The expression of HAND2‐AS1 in cervical cancer and normal samples from TCGA database. C, The expression of HAND2‐AS1 in cervical cancer tissues (n = 57) and normal tissues (n = 20) examined by RT‐qPCR. D, The relationship between HAND2‐AS1 expression and OS of patients analysed by the Kaplan‐Meier method. E, The expression of HAND2‐AS1 in human cervical epithelial immortalized cell H8 and cervical cancer cells Caski, HeLa, Siha and HCE1 examined by RT‐qPCR. *P < .05. The values in (D) were enumeration data, which were expressed as percentage, and other data were measurement data, which were expressed as mean ± standard deviation. Data between two groups were compared by independent sample *t* test, and data among multiple groups were analysed by one‐way analysis of variance, with Tukey's post hoc test conducted. The Kaplan‐Meier method was applied for calculating the OS of patients, and univariate analysis was performed using log‐rank test. Cell experiment was repeated 3 times. HAND2‐AS1, heart‐ and neural crest derivative‐expressed protein 2‐AS1; TCGA, The Cancer Genome Atlas; RT‐qPCR, reverse transcription quantitative‐polymerase chain reaction; OS, overall survival; DEGs, differentially expressed genes

Microarray data (GSE63678) were initially used to identify the cervical cancer‐related differentially expressed genes (DEGs), and lncRNA HAND2‐AS1 was observed to express at a relatively poor level in cervical cancer compared with the normal samples (Figure 1A). In addition, it was found from TCGA that lncRNA HAND2‐AS1 was down‐regulated in cervical cancer relative to normal samples (Figure 1B). RT‐qPCR was applied for detection of HAND2‐AS1 expression in 57 cervical cancer tissues and normal tissues. The results in Figure 1C revealed a significant down‐regulation in HAND2‐AS1 in cervical cancer tissues in comparison with the normal tissues (*P* < .05). Further analysis of cervical cancer specimens demonstrated that HAND2‐AS1 was negatively correlated with Federation International of Gynecology and Obstetrics (FIGO) staging, differentiation and lymph node metastasis of patients, while there was an insignificant correlation with the patients' age, tumour diameter or pathology subtypes (Table 2). Subsequently, the Kaplan‐Meier method was used to determine the relation between HAND2‐AS1 expression and OS of patients, which found a poor OS in cervical cancer patients with reduced HAND2‐AS1 expression compared with the patients with elevated HAND2‐AS1 expression (Figure 1D; log‐rank *P* < .05). Next, the prognostic factors of cervical cancer (Table S1) were analysed by Cox regression model. Multivariate Cox regression analysis showed that FIGO stage, lymph node metastasis and HAND2‐AS1 expression were all independent prognostic indicators in patients with cervical cancer, indicating that the expression of HAND2‐AS1 was related to the poor prognosis of cervical cancer. Furthermore, 4 cervical cancer cell lines (Caski, HeLa, Siha and HCE1) and the normal human cervical epithelial cells H8 were collected in order to determine HAND2‐AS1 expression using RT‐qPCR. As shown in Figure 1E, the expression of HAND2‐AS1 was remarkably lower in cervical cancer cells than that in H8 cells (*P* < .05). In addition, HeLa cells presented with the lowest expression of HAND2‐AS1 among the four cervical cancer cell lines; thus, HeLa cells were selected for subsequent experiments. Therefore, these findings provided evidence that reduced the expression of HAND2‐AS1 may be involved in the malignant progression of cervical cancer.

**Table 2:** Expression of HAND2‐AS1 and its correlation with clinicopathological parameter of patients with cervical cancer

Characteristics	Case	HAND2‐AS1 expression		*P*
		High	Low	
Age (y)				.141
≥49	41	18	23	
<49	16	11	5	
FIGO stage				.025
I	22	9	13	
II	20	15	5	
III	15	5	10	
Differentiation				.007
Well‐differentiated	22	14	8	
Moderately differentiated	14	10	4	
Poorly differentiated	21	5	16	
Tumour diameter (cm)				.408
≥4	19	8	11	
<4	38	21	17	
Lymph node metastasis				.019
Yes	16	4	12	
No	41	25	16	
Pathological type				.783
Adenocarcinoma	19	9	10	
Squamous cell carcinoma	38	20	18	

Note

Chi‐square test was used for analysis.

### 3.2 Overexpression of HAND2‐AS1 suppresses cervical cancer proliferation, migration and invasion

**Figure 2:**
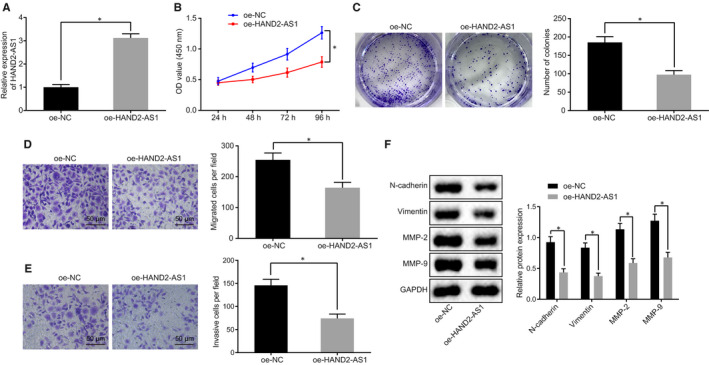
HAND2‐AS1 represses the cell proliferation, migration and invasion of cervical cancer cells. A, The expression of HAND2‐AS1 in cervical cancer cells transfected with oe‐HAND2‐AS1 or oe‐NC plasmid detected by RT‐qPCR; *P < .05. B, The cell proliferation of cervical cancer cells examined by CCK‐8 assay; *P < .05. C, The ability of colony formation of cervical cancer cells; *P < .05. D, E, the migration and invasion of cervical cancer cells examined by Transwell assay (×200, scale bar = 50 μm); *P < .05. F, The protein expression of N‐cadherin, vimentin, MMP‐2 and MMP‐9 in cervical cancer cells determined by Western blot analysis; *P < .05. The results data were measurement data, which were presented as mean ± standard deviation. Data that conformed to normal distribution and homogeneity of variance were compared by unpaired *t* test between two groups. The repeated‐measures ANOVA was applied for comparing the data among multiple groups at different time‐points, and the Bonferroni's post hoc test was conducted. Cell experiment was repeated 3 times. RT‐qPCR, reverse transcription quantitative‐polymerase chain reaction; HAND2‐AS1, heart‐ and neural crest derivative‐expressed protein 2‐AS1; oe, overexpression; NC, negative control; CCK‐8, Cell Counting Kit‐8; MMP, matrix metallopeptidase; ANOVA, analysis of variance

The proliferation, invasion and migration of HeLa cells were determined in order to investigate the role HAND2‐AS1 played in modulating the malignant phenotype of cervical cancer cells. To achieve this, oe‐HAND2‐AS1 was transfected into HeLa cells, and the robustly elevated expression of HAND2‐AS1 was detected using RT‐qPCR compared with the cells delivered with oe‐NC (Figure 2A; *P* < .05). Next, CCK‐8 assay and colony formation assay were conducted to determine the proliferation of HeLa cells. As shown in Figure 2B,C, HAND2‐AS1 overexpression significantly attenuated the proliferation of HeLa cells compared with oe‐NC group (*P* < .05). Next, cell migration and invasion were detected with the help of the Transwell assay, and the migration and invasion significantly reduced in HeLa cells with overexpressed HAND2‐AS1 relative to the cells in oe‐NC group (Figure 2D,E; *P* < .05). Furthermore, the expression of N‐cadherin, vimentin, MMP‐2 and MMP‐9 was analysed by Western blots. As shown in Figure 2F, there was a significant suppression in the expression of N‐cadherin, vimentin, MMP‐2 and MMP‐9 in HeLa cells overexpressing HAND2‐AS1 compared with cells in the oe‐NC group (*P* < .05). Collectively, the above results revealed that HAND2‐AS1 repressed proliferation, migration and invasion of cervical cancer cells.

### 3.3 HAND2‐AS1 interacts with transcription factor E2F4 at the promoter of C16orf74

**Figure 3:**
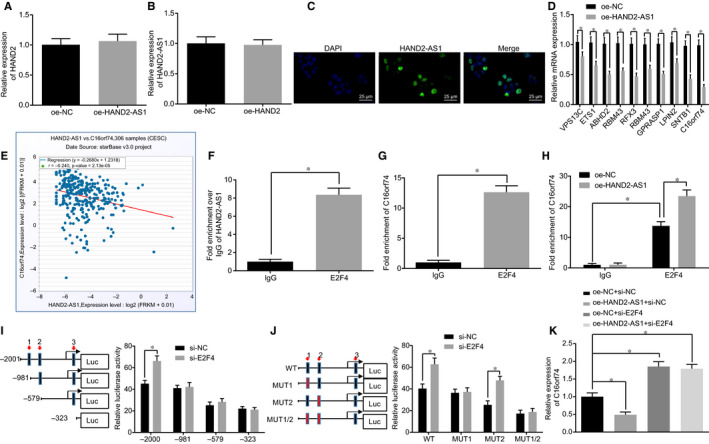
HAND2‐AS1 reduces C16orf74 expression through recruiting E2F4. A, HAND2 mRNA expression in cells transfected with oe‐HAND2‐AS1 or oe‐NC examined by RT‐qPCR. B, HAND2‐AS1 expression in cells transfected with oe‐HAND2 or oe‐NC examined by RT‐qPCR. C, The subcellular localization of HAND2‐AS1 in cells examined by FISH assay (×400, scale bar = 25 μm). D, The expression of the potential target genes in cells transfected with oe‐HAND2‐AS1 or oe‐NC plasmids detected by RT‐qPCR. E, The co‐expression of the HAND2‐AS1 with the C16orf74, the *X*‐axis represents the expression of the HAND2‐AS1, the *Y*‐axis represents the expression of the C16orf74, each point in the Figure represents a sample, and the *P* value and the correlation coefficient *R* are located at the upper left. F, The binding between HAND2‐AS1 and E2F4 measured by RIP assay. G, The binding between E2F4 and C16orf74 was examined by ChIP assay. H, The enrichment of C16orf74 promoter by E2F4 in cells transfected with oe‐HAND2‐AS1 or oe‐NC plasmids. I, J, Dual‐luciferase reporter gene assay verifying the binding relationship between wild‐type or mutated E2F4 and C16orf74 promoter. K, The mRNA expression of C16orf74 in cells transfected with HAND2‐AS1 and/or E2F4 siRNA detected by RT‐qPCR. *P < .05. The result data were measurement data and were expressed as mean ± standard deviation. Data that conformed to normal distribution and homogeneity of variance were compared by unpaired *t* test between two groups. Data among multiple groups were compared using one‐way ANOVA, with Tukey's post hoc test conducted. Cell experiment was repeated 3 times. HAND2‐AS1, heart‐ and neural crest derivative‐expressed protein 2‐AS1; FISH, fluorescence in situ hybridization; RT‐qPCR, reverse transcription quantitative‐polymerase chain reaction; RIP, RNA immunoprecipitation; CCHP. chromatin immunoprecipitation; oe, overexpression; NC, negative control; ANOVA, analysis of variance; C16orf74, chromosome 16 open reading frame 74

In order to examine whether HAND2‐AS1 regulated the transcription of HAND2 in cervical cancer cells, HeLa cells were transfected with oe‐HAND2‐AS1 and the mRNA expression of HAND2 was determined. As shown in Figure 3A, the overexpression of HAND2‐AS1 had little effect on HAND2 mRNA expression (*P* > .05). Conversely, the mRNA expression of HAND2‐AS1 was not influenced by HAND2 overexpression (Figure 3B; *P* > .05), suggesting that upstream HAND2‐AS1 did not regulate the HAND2 transcription in HeLa cells. At the same time, FISH assay was carried out to determine subcellular localization of HAND2‐AS1 in HeLa cells, which found that HAND2‐AS1 was mainly located in nucleus (Figure 3C). LncMAP (tp://bio‐bigdata.hrbmu.edu.cn/LncMAP/) was applied to predict the potential mechanism of HAND2‐AS1 in cervical cancer (Table 3). RT‐qPCR was conducted to examine the expression of the top 10 potential target genes in HeLa cells transfected with oe‐AND2‐AS1. As shown in Figure 3D, all 10 genes were found to be down‐regulated and C16orf74 exhibited the largest reduction (*P* < .05). The co‐expression of HAND2‐AS1 and C16orf74 in TCGA was further analysed, and it was found that there was a significant negative correlation between HAND2‐AS1 and C16orf74 (Figure 3E).

**Table 3:** The potential transcription factors and downstream target genes that predicted to be bound with HAND2‐AS1, by LncMAP

LncRNA symbol	TF symbol	Gene symbol
HAND2‐AS1	REST	VPS13C
HAND2‐AS1	REST	ETS1
HAND2‐AS1	REST	ABHD2
HAND2‐AS1	REST	RBM43
HAND2‐AS1	EP300	RFX3
HAND2‐AS1	TCF12	RBM43
HAND2‐AS1	TAF1	GPRASP1
HAND2‐AS1	RXRA	LPIN2
HAND2‐AS1	RXRA	SNTB1
HAND2‐AS1	E2F4	C16orf74

Abbreviations: LncRNA, long non‐coding RNA; TF, transcription factor.

The underlying mechanism by which C16orf74 is involved in cervical cancer was further investigated. LncMAP analysis was performed, and E2F4 was found to be the potential transcription factor that mediated the modulation of HAND2‐AS1 on C16orf74 expression (Table 3). The binding site between HAND2‐AS1 and E2F4 was verified with the use of the RIP assay, and HAND2‐AS1 was detected in E2F4 immunoprecipitated complex, suggesting that HAND2‐AS1 bound to E2F4 (Figure 3F; *P* < .05). Moreover, ChIP assay was performed, and the binding between E2F4 and C16orf74 was substantiated (Figure 3G; *P* < .05). In addition, the overexpression of HAND2‐AS1 enhanced the binding between E2F4 and C16orf74 (Figure 3H; *P* < .05). These data strengthened the possibility that HAND2‐AS1 could regulate C16orf74 expression by recruiting E2F4.

The potential binding sites of E2F4 on C16orf74 promoter region were predicted in Jaspar (http://jaspar.genereg.net/), and three potential sites were identified (Table 4). Next, the sequences were truncated or mutated, and subjected to dual‐luciferase reporter gene assay. As shown in Figure 3I,J, upon truncation or mutation on site 1, the ability of si‐E2F4 to activate C16orf74 was significantly decreased when compared with si‐NC (*P* < .05), while the ability of E2F4 to inhibit C16orf74 was not significantly affected following mutation of other sites (*P* > .05), suggesting that the site 1 was the main site for transcription factor E2F4 that can inhibit the transcriptional activity of C16orf74. Lastly, HeLa cell was transfected with oe‐HAND2‐AS1 and E2F4 siRNA, and the expression of C16orf74 was detected by RT‐qPCR. The mRNA of C16orf74 was significantly decreased in cells upon HAND2‐AS1 overexpression, which could be reversed by the knock‐down of E2F4 (Figure 3J; *P* < .05). Therefore, our data suggest that HAND2‐AS1 might regulate C16orf74 expression by recruiting E2F4 to the promoter of C16orf74.

**Table 4:** The potential binding sites of transcription E2F4 on C16orf74 promoter region analysed by JASPAR website

Matrix ID	Name	Score	Relative score	Sequence ID	Start	End	Strand	Predicted sequence
MA0470.1	E2F4	12.083339	0.9361628	C16orf74	981	991	+	ccgcgggaggc
MA0470.1	E2F4	10.722039	0.914605213	C16orf74	579	589	+	gggcggggggg
MA0470.1	E2F4	9.307395	0.892202857	C16orf74	323	333	+	ccgcggggagg

### 3.4 Increased occupancy of E2F4 at the promoter of C16orf74 mediated by HAND2‐AS1 inhibits cervical cancer progression

**Figure 4:**
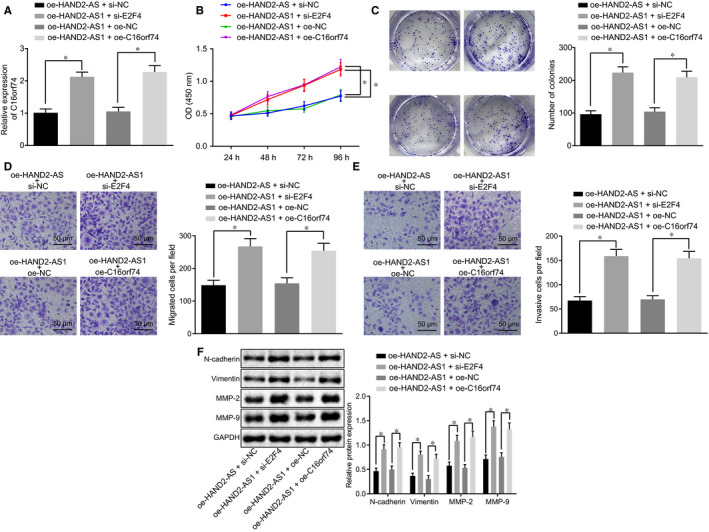
HAND2‐AS1 blocks the proliferation, migration and invasion of cervical cancer cells through suppression of C16orf74 expression via E2F4. A, The mRNA expression of C16orf74 in cells transfected with oe‐C16orf74 or si‐E2F4 plasmids in the presence of oe‐HAND2‐AS1 detected by RT‐qPCR. *P < .05. B‐E, the cell proliferation (B), colony formation (C), migration (D) (×200, scale bar = 50 μm) and invasion (E) (×200, scale bar = 50 μm) of cervical cancer cells transfected with oe‐C16orf74 or si‐E2F4 plasmids in the presence of oe‐HAND2‐AS1 examined by CCK‐8 assay, colony formation assay and Transwell assay, respectively; *P < .05. F, The expression of N‐cadherin, vimentin, MMP‐2 and MMP‐9 in cervical cancer cells transfected with HAND2‐AS1 and E2F4 siRNA or C16orf74 examined by Western blot analysis; *P < .05. The result data were measurement data and were expressed as mean ± standard deviation. Data that conformed to normal distribution and homogeneity of variance were compared by unpaired *t* test between two groups. The repeated measurement ANOVA was applied for comparing the data among multiple groups at different time‐points, and the Bonferroni's post hoc test was conducted. Cell experiment was repeated 3 times. RT‐qPCR, reverse transcription quantitative‐polymerase chain reaction; HAND2‐AS1, heart‐ and neural crest derivative‐expressed protein 2‐AS1; oe, overexpression; NC, negative control; CCK‐8, Cell Counting Kit‐8; MMP, matrix metallopeptidase; ANOVA, analysis of variance; C16orf74, chromosome 16 open reading frame 74

Furthermore, the effect of HAND2‐AS1/E2F4/C16orf74 axis on the progression of cervical cancer was explored. Firstly, RT‐qPCR was conducted to examine the effects of E2F4 on the expression of C16orf74 in cervical cancer cells, and the result showed a progressive elevation in the expression of C16orf74 in HAND2‐AS1‐overexpressed cervical cancer cells upon E2F4 silencing, while the same trend was observed in cells with C16orf74 and HAND2‐AS1 overexpression (Figure 4A; *P* < .05). Next, we investigated whether HAND2‐AS1/E2F4/C16orf74 regulated the malignant characteristic of cervical cancer cells. We found that upon HAND2‐AS1 overexpression, E2F4 silencing or C16orf74 overexpression could promote the proliferation of cervical cancer cells (Figure 4B; *P* < .05). In addition, we observed that the ability of colony formation in cervical cancer cells was increased upon E2F4 silencing or C16orf74 overexpression in the presence of HAND2‐AS1 overexpression (Figure 4C; *P* < .05). In addition, cell migration and invasion were also enhanced following E2F4 silencing or C16orf74 overexpression treatment in cervical cancer cell overexpression of HAND2‐AS1 (Figure 4D,E; *P* < .05). Furthermore, Western blot analysis was used to detect the expression of N‐cadherin, vimentin, MMP‐2 and MMP‐9 in cervical cancer cells, the results of which showed an up‐regulation in these proteins upon E2F4 silencing or C16orf74 overexpression in the presence of HAND2‐AS1 overexpression (Figure 4F; *P* < .05). The aforementioned findings led to a conclusion that HAND2‐AS1 overexpression exerted inhibitory effect on proliferation, migration and invasion of cervical cancer cells via repression of C16orf74 through the up‐regulation of E2F4.

### 3.5 Overexpression of HAND2‐AS1 inhibits the tumorigenesis of cervical cancer in vivo through suppression of C16orf74 via recruitment of E2F4

**Figure 5:**
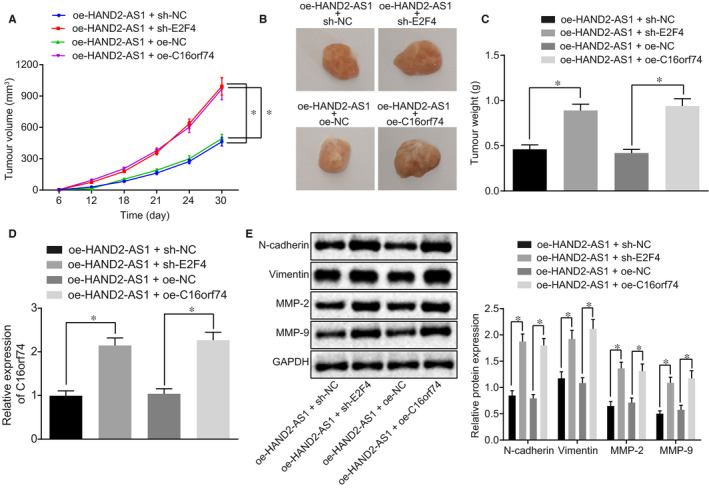
HAND2‐AS1 represses the tumorigenesis of cervical cancer in vivo through C16orf74 and E2F4. A‐C, the tumour volume and weight in nude mice bearing the HAND2‐AS1 overexpressed cells upon E2F4 knock‐down or C16orf74 overexpression; *P < .05. D, The mRNA expression of C16orf74 in tumour tissues was detected by RT‐qPCR; *P < .05. E, The protein expression of N‐cadherin, vimentin, MMP‐2 and MMP‐9 in tumour tissues of mice examined by Western blot analysis; *P < .05. The result data were measurement data and were expressed as mean ± standard deviation. Data that conformed to normal distribution were compared by unpaired *t* test between two groups. The repeated‐measures ANOVA was applied for comparing the data among multiple groups at different time‐points, and the Bonferroni's post hoc test was conducted. n = 12. RT‐qPCR, reverse transcription quantitative‐polymerase chain reaction; HAND2‐AS1, heart‐ and neural crest derivative‐expressed protein 2‐AS1; oe, overexpression; NC, negative control; MMP, matrix metallopeptidase; ANOVA, analysis of variance; C16orf74, chromosome 16 open reading frame 74

Cervical cancer cells treated with designated plasmids were delivered into nude mice, and the tumour tissue growth was recorded. The results in Figure 5A revealed that compared with the control mice (oe‐HAND2‐AS1 + sh‐NC or oe‐NC), the tumour in mice with E2F4 silencing or C16orf74 overexpression exhibited a faster growth rate (*P* < .05). Consistently, tumour size and weight in mice harbouring HAND2‐AS1 overexpression and E2F4 silencing or C16orf74 overexpression were markedly increased relative to the control mice (Figure 5B,C; *P* < .05). Moreover, RT‐qPCR was conducted, and the enhanced expression of C16orf74 was detected in the tumours with HAND2‐AS1 overexpression and E2F4 silencing or C16orf74 overexpression (Figure 5D; *P* < .05), further supporting the critical role of C16orf74 expression in the cervical tumour growth. The protein expression of N‐cadherin, vimentin, MMP‐2 and MMP‐9 was also analysed in tumour tissues in our established xenograft model. As shown in Figure 5E, the protein expression of N‐cadherin, vimentin, MMP‐2 and MMP‐9 was profoundly elevated in tumours of nude mice injected with oe‐HAND2‐AS1 + sh‐E2F4 and oe‐HAND2‐AS1 + oe‐C16orf74 (*P* < .05). Overall, our findings demonstrate that HAND2‐AS1 activated E2F4 to down‐regulate C16orf74 in cervical cancer.

## 4 DISCUSSION

Various therapies have been applied to manage different subtypes of cervical cancer; however, adverse effects were inevitable for some of these therapies, an example of which is vaginal fibrosis and dyspareunia induced by radiotherapy.^24^ Therefore, the pathogenesis of cervical cancer needs to be explored further to discover more therapeutic targets for cervical cancer treatment. LncRNAs have been found to participate in tumorigenesis and play significant roles in cervical cancer.^25^, ^26^ E2F4 was identified to be critical in the regulation of cell progression in various cancers and found to be related to cervical cancer.^16^ We verified the hypothesis that lncRNA HAND2‐AS1 might participate in the cervical cancer physiopathology. Our findings indicated that HAND2‐AS1 suppressed the cell proliferation, migration, invasion and tumorigenesis of cervical cancer by recruiting E2F4 to down‐regulate C16orf74 expression.

Firstly, HAND2‐AS1 was found to be poorly expressed in cervical cancer tissue and cells, which were consistent with previous reports, suggesting that HAND2‐AS1 was down‐regulated in various cancers, such as colorectal cancer (CRC) and non–small‐cell lung cancer.^27^, ^28^ LncRNAs acted as a hinge to recruit transcription factors to gene promoter,^19^ and we also found that HAND2‐AS1 could bind with transcription factor E2F4 and recruit E2F4 to inhibit the expression of C16orf74.

Furthermore, up‐regulation of HAND2‐AS1 was found to suppress cell proliferation, migration, invasion and tumorigenesis of cervical cancer. HAND2‐AS1 restoration resulted in reduced N‐cadherin, vimentin, MMP‐2 and MMP‐9 expression, which was consistent with a former study in which HAND2‐AS1 reduced MMP‐2 and MMP‐9 expressions in chronic myeloid leukaemia.^29^ Moreover, increased N‐cadherin and vimentin expression has been linked with high cell migration in tumours,^30^ further supporting the migration‐inhibitory role of HAND2‐AS1 in cervical cancer. Importantly, the overexpression of HAND2‐AS1 resulted in the suppression in cell migration and invasion in endometrioid endometrial carcinoma (EEC), indicating that HAND2‐AS1 played a role as a promising biomarker for EEC.^31^ Furthermore, up‐regulated E2F4 led to the suppression of cell proliferation and tumorigenesis of Burkitt lymphoma.^15^ LncRNAs have been demonstrated to mediate gene expression at transcriptional, post‐transcriptional or epigenetic levels.^32^ The silencing of E2F4 diminished proliferation of human intestinal epithelial cells and CRC cells,^33^ which supports the regulatory role of E2F4 in cell progression in cancers. Furthermore, up‐regulation of C16orf74 was found in human PDAC, and down‐regulation of C16orf74 or cell‐permeable peptide DN‐C16orf74 led to the suppression of the proliferation and invasion of pancreatic cells, exerting tumour‐suppressive functions.^34^ It is worth noting that the endogenous threonine 44‐phosphorylated form of C16orf74 interacted with the protein phosphatase 3 catalytic subunit alpha (PPP3CA) in the PPP3CA‐binding motif in PDAC cells, and the overexpression of mutants of C16orf74 lacking the PDIIIT sequence repressed the invasive activity relative to wild‐type C16orf74, implying that this link was indispensable for PDAC cell invasion^18^ C16orf74 has been found to be positively correlated with PRSS3 in bladder cancer, and this relationship might act as an instrumental role in promoting its invasiveness.^17^ C16orf74 is found in its homodimer form under the cell membrane and binds to integrin αVβ3 and regulates cell invasion by activating Rho family and MMP2 in PDAC.^35^ Other mechanisms by which C16orf74 promotes cancer development remained unknown. However, we provide evidence that HAND2‐AS1 recruited E2F4 to modulate C16orf74 expression. We also demonstrated that up‐regulation of C16orf74 can rescue the inhibitory role HAND2‐AS1 overexpression has on cervical cancer cell proliferation, migration and invasion, as evidenced by promoted expression of N‐cadherin, vimentin, MMP‐2 and MMP‐9.

**Figure 6:**
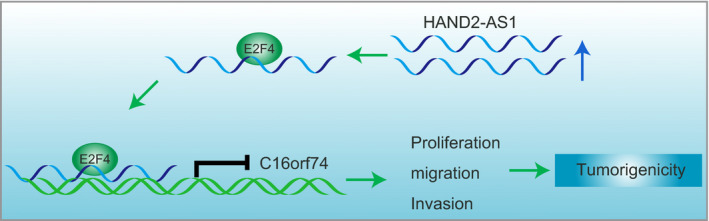
The molecular mechanism involving HAND2‐AS1/E2F4/C16orf74 axis in regulating the progression of cervical cancer. HAND2‐AS1 was poorly expressed in cervical cancer and could bind to the transcription factor E2F4. Significantly, HAND2‐AS1 overexpression recruited E2F4 to the C16orf74 promoter region and down‐regulated the level of C16orf74, thus suppressing cell proliferation, migration and invasion of cervical cancer. HAND2‐AS1, heart‐ and neural crest derivative‐expressed protein 2‐AS1; C16orf74, chromosome 16 open reading frame 74

Collectively, the findings obtained from this study further elucidated the significant role of HAND2‐AS1 in regulating the development of cervical cancer. HAND2‐AS1 attenuated cell proliferation, migration, invasion and tumorigenesis of cervical cancer by down‐regulating C16orf74 expression through recruiting E2F4 (Figure 6). These findings can aid the development of a novel therapeutic option for cervical cancer.

## CONFLICTS OF INTEREST

None.

## Supporting information

Table S1

## Data Availability

Data generated and analysed as part of this study are included in the manuscript or are available upon request from the corresponding author.
