# Supplementary material for: LncRNA HAND2‐AS1 represses cervical cancer progression by interaction with transcription factor E2F4 at the promoter of C16orf74
Source: J Cell Mol Med. 2020 Apr 21;24(11):6015–27. doi: 10.1111/jcmm.15117 (PMC7294116; doi:10.1111/jcmm.15117)
Supplement: Supplementary file 1 — Table S1 [file JCMM-24-6015-s001.docx]

**Supplementary Table S1** Univariate and multivariate analysis of OS in the whole series

| Variables | Univariate analysis | | Multivariate analysis | |
| --- | --- | --- | --- | --- |
|  | HR (95%CI) | *p* | HR (95%CI) | *p* |
| Age (≥ 49 vs < 49) | 0.901 (0.367, 2.215) | 0.821 | 0.548 (0.194, 1.551) | 0.257 |
| FIGO stage (Ⅰ vs Ⅱ vs Ⅲ) | 2.385 (1.347, 4.222) | 0.003 | 2.345 (1.140, 4.826) | 0.021 |
| Differentiation (Well vs moderately vs poorly) | 1.415 (0.859, 2.330) | 0.173 | 1.950 (0.853, 4.459) | 0.114 |
| Tumor diameter (≥ 4 vs < 4) | 1.383 (0.590, 3.240) | 0.455 | 1.431 (0.275, 6.446) | 0.205 |
| LN involvement (Yes vs No) | 4.907 (2.072, 11.619) | 0.000 | 5.874 (1.376, 25.079) | 0.017 |
| Pathological type (Adenocarcinoma vs squamous cell carcinoma) | 0.720 (0.308, 1.688) | 0.450 | 0.430 (0.117, 1.584) | 0.722 |
| HAND2-AS1 expression (Positive versus Negative) | 0.089 (0.026, 0.304) | 0.000 | 0.067 (0.011, 0.394) | 0.003 |
